# Can an incomplete ERAS protocol reduce postoperative complications compared with conventional care in laparoscopic radical resection of colorectal cancer? A multicenter observational cohort and propensity score-matched analysis

**DOI:** 10.3389/fsurg.2022.986010

**Published:** 2022-08-26

**Authors:** Chenxing Jian, Zili Zhou, Shen Guan, Jianying Fang, Jinhuang Chen, Ning Zhao, Haijun Bao, Xianguo Li, Xukai Cheng, Wenzhong Zhu, Chunkang Yang, Xiaogang Shu

**Affiliations:** ^1^ Department of Gastrointestinal Surgery and Department of Emergency Surgery, Union Hospital, Tongji Medical College, Huazhong University of Science and Technology, Wuhan, China; ^2^ Department of Minimally Invasive Surgery, Affiliated Hospital of Putian University, Teaching Hospital of Fujian Medical University, Putian, China; ^3^ Department of Gastrointestinal Surgery, Sichuan Provincial People's Hospital, University of Electronic Science and Technology of China, Chengdu, China; ^4^ Department of Gastrointestinal Surgical Oncology, Fujian Cancer Hospital and Fujian Medical University Cancer Hospital, Fuzhou, China

**Keywords:** incomplete ERAS protocol, colorectal cancer, traditional care, complications, propensity score-matched analysis

## Abstract

**Background:**

The patients undergoing laparoscopic radical colorectomy in many Chinese hospitals do not achieve high compliance with the ERAS (enhanced recovery programs after surgery) protocol.

**Methods:**

The clinical data from 1,258 patients were collected and divided into the non-ERAS and incomplete ERAS groups.

**Results:**

A total of 1,169 patients were screened for inclusion. After propensity score-matched analysis (PSM), 464 pairs of well-matched patients were generated for comparative study. Incomplete ERAS reduced the incidence of postoperative complications (*p* = 0.002), both mild (6.7% vs. 10.8%, *p* = 0.008) and severe (3.2% vs. 6.0%, *p* = 0.008). Statistically, incomplete ERAS reduced indirect surgical complications (27,5.8% vs. 59, 12.7) but not local complications (19,4.1% vs. 19, 4.1%). The subgroup analysis of postoperative complications revealed that all patients benefited from the incomplete ERAS protocol regardless of sex (male, *p* = 0.037, 11.9% vs. 17.9%; female, *p* = 0.010, 5.9% vs. 14.8%) or whether neoadjuvant chemotherapy was administered (neoadjuvant chemotherapy, *p* = 0.015, 7.4% vs. 24.5%; no neoadjuvant chemotherapy, *p* = 0.018, 10.2% vs. 15.8%). Younger patients (<60 year, *p* = 0.002, 7.6% vs. 17.5%) with a low BMI (<22.84, 9.4% vs. 21.1%, *p* < 0.001), smaller tumor size (<4.0 cm, 8.1% vs. 18.1%, *p* = 0.004), no fundamental diseases (8.8% vs. 17.0%, *p* = 0.007), a low ASA score (1/2, 9.7% vs. 16.3%, *p* = 0.004), proximal colon tumors (ascending/transverse colon, 12.2% vs. 24.3%, *p* = 0.027), poor (6.1% vs. 23.7%, *p* = 0.012)/moderate (10.3% vs. 15.3%, *p* = 0.034) tumor differentiation and no preoperative neoadjuvant radiotherapy (10.3% vs. 16.9%, *p* = 0.004) received more benefit from the incomplete ERAS protocol.

**Conclusion:**

The incomplete ERAS protocol decreased the incidence of postoperative complications, especially among younger patients (<60 year) with a low BMI (<22.84), smaller tumor size (<4.0 cm), no fundamental diseases, low ASA score (1/2), proximal colon tumors (ascending/transverse colon), poor/moderate differentiation and no preoperative neoadjuvant radiotherapy. ERAS should be recommended to as many patients as possible, although some will not exhibit high compliance. In the future, the core elements of ERAS need to be identified to improve the protocol.

## Introduction

Since the ERAS (enhanced recovery programs after surgery) protocol was first recommended, it has gained widespread popularity as a common concept in different professional fields (1–11). During the past decade, many surgeons in China have also used ERAS. Some studies have reported perioperative benefits in patients who are highly compliant with the ERAS protocol (12–14). However, ERAS is not used for all patients in many Chinese hospitals, and some patients do not achieve high compliance with the protocol. This is common among patients not included in research studies, as most studies focus on highly compliant ERAS patients and seem to ignore the nonadherent subset of this population. Our previous study showed that ERAS failure occurred in 38 (17.9%) patients undergoing ERAS among 212 selected gastric cancer patients because of the following factors: advanced age, high ASA score, lack of preoperative education and combined surgery (15). The results of that study indicated that even some select ERAS patients failed to adhere to ERAS. In that study, ERAS failure was defined as more than 7 days of hospitalization due to postoperative complications, unplanned readmission or death within 30 days of surgery. If the program is extended to all patients, the failure rate will be even higher. Other researchers have also reported such failures after colorectal cancer surgery. Studies conducted by Korean researchers on ERAS in colorectal cancer patients have reached similar conclusions (16, 17). Therefore, a new question arises: Do such cases of failure still confer some advantage over no ERAS protocol? To answer this question, we collected more case data and conducted this retrospective cohort comparison study using PSM. To the best of our knowledge, few studies have involved large volumes of patients with low compliance to ERAS protocols versus traditional care. In this study, similar to many researchers (18–20), incomplete ERAS was defined as compliance <70%.

The aim of this study (Research Registration Unique Identifying Number: clinicaltrials NCT05412355) was to evaluate the perioperative complications of incomplete ERAS in patients undergoing laparoscopic radical colorectomy by a multicenter propensity-matched analysis. In addition, we analyzed the impact of incomplete ERAS in subgroups of patients with different clinicopathological characteristics.

## Methods

### Compliance calculation

Compliance scores were calculated according to the ERAS protocol for colorectal cancer, as shown in Table 1. We calculated the compliance score for each patient by reviewing the compliance of each of the 20 nodes of the protocol, with a score of 5 for a node that was fully complied with and 0 for a node that was not.

**Table 1:** ERAS protocols for colorectal cancer surgery^a^.

PreOp	Preoperative education, carbohydrate loading, bowel preparation, preoperative nutritional evaluation, dietary plan, prophylactic antibiotic.
Intraoperative	Minimally invasive surgery, nasogastric tube, restricted infusion, body temperature during surgery, abdominal drainage.
1st PostOp Day	Pain relief, removal of the catheter, postoperative liquid diet.
2nd PostOp Day	Early mobilization, intestinal recovery.
3rd PostOp Day	Stop analgesic pump, postoperative soft food.
4th PostOp Day	Postoperative solid food.
5th PostOp Day	Check discharge criteria.

^a^ Twenty nodes were used to calculate the compliance score (5 points per node for a total of 100 points) for each case.

### Patient selection and study design

The clinical data of 1,258 patients undergoing laparoscopic radical colorectomy at four large university hospitals between August 2008 and December 2020 were collected for this study. After excluding cases that did not meet the criteria for inclusion, eligible cases were divided into the non-ERAS and incomplete ERAS groups. The ERAS Society Guidelines for colorectal resection were established in 2012, and since then, ERAS has been gradually accepted by Chinese surgeons. The non-ERAS group included patients who had never completed an ERAS protocol, most of whom were hospitalized from 2008 to 2012. The incomplete ERAS group included patients who had ERAS compliance <70% and were hospitalized from 2012 to 2020. The inclusion criteria were 1. age ≥18 years; 2. pathological diagnosis of colorectal cancer; and 3. radical laparoscopic surgery was performed. The exclusion criteria were 1. ERAS compliance ≥70%; 2. open surgery; 3. conversion to laparotomy after laparoscopic surgery; 4. severe mental illness; 5. pregnancy or lactation; 6. simultaneous malignant tumors in multiple organs; 7. history of other malignant tumors; and 8. emergency radical colorectomy. All included cases were completed after the surgeon mastered the learning curve, and the surgical technique and skills of the surgeon at each institution were recognized and confirmed by the surgeons at the other institutions. Postoperative complications were defined as surgery-related complications during the postoperative hospital stay and surgery-related readmission or death within 30 days after surgery.

### Matching

PSM was used to balance the following factors between the two groups: sex, age, BMI (body mass index), fundamental diseases, ASA score (American Society of Anesthesiologists ASA score), tumor location, tumor size, tumor differentiation, perineural invasion, lymphovascular invasion, tumor stage, and neoadjuvant chemoradiotherapy. After calculating the propensity score for each patient, we matched the patients 1:1 with an SD set caliper width of 0.01. Some items in the clinical data of the two groups showed significant differences before PSM (shown in Table 1), but they were adjusted and confirmed to be balanced after matching (Table 2).

**Table 2:** Characteristics of all patients before matching.

	Incomplete ERAS (*n* = 650)		Non-ERAS (*n* = 519)		*p*
	*N*	%	*N*	%	
Compliance with ERAS (midian + IQR^a^)	50% (45%–55%)				
Age	60.77 ± 11.61		58.88 ± 12.88		0.009
BMI	23.12 ± 3.06		22.64 ± 3.28		0.009
Tumor size	4.17 ± 2.18		4.37 ± 1.88		0.098
Sex					
Male	422	64.9	340	65.5	0.834
Female	228	35.1	179	34.5	
Fundamental disease					0.690
Yes	327	50.3	255	49.1	
No	323	49.7	264	50.9	
ASA score					<0.001
1	11	1.7	98	18.9	
2	580	89.2	386	74.4	
3	58	8.9	34	6.6	
4	1	0.2	1	0.2	
Tumor location^b^					0.020
1	127	19.5	91	17.5	
2	23	3.5	17	3.3	
3	69	10.6	30	5.8	
4	123	18.9	97	18.7	
5	308	47.4	284	54.7	
Neoadjuvant radiotherapy					<0.001
Yes	69	10.6	25	4.8	
No	581	89.4	494	95.2	
Neoadjuvant chemotherapy					0.003
Yes	106	16.3	53	10.2	
No	544	83.7	466	89.8	
Differentiation					0.622
Low	72	11.1	64	12.3	
Moderate	553	85.1	431	83.0	
High	25	3.8	24	4.6	
Perineural invasion					0.266
Yes	128	19.7	89	17.1	
No	522	80.3	430	82.9	
Lymphovascular invasion					<0.001
Yes	250	38.5	139	26.8	
No	400	61.5	380	73.2	
TNM stage					0.173
0	18	2.8	7	1.3	
1	106	16.3	75	14.5	
2	214	32.9	173	33.3	
3	245	37.7	221	42.6	
4	67	10.3	43	8.3	
Postoperative complications					0.001
Yes	64	9.8	85	16.4	
No	586	90.2	434	83.6	

^a^ IQR, interquartile range.

^b^ 1: ascending colon; 2: transverse colon; 3: descending colon; 4: sigmoid colon; 5: rectum.

### Statistical analysis

SPSS 25.0 software was used for analysis. Categorical and continuous variables were compared with the *x*^2^ test and Student's t-test, respectively. For subgroup analysis, Fisher's exact test was used to analyze two factors because of the small number of cases. A *p* value < 0.05 was considered statistically significant.

## Results

### Patient characteristics

**Figure 1:**
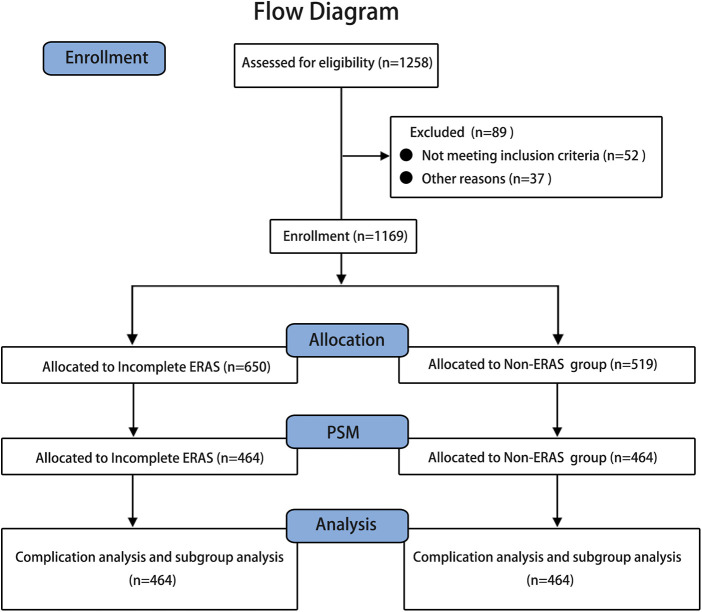
Flow diagram the clinical data of 1,258 patients undergoing laparoscopic radical colorectomy at four large university hospitals between August 2008 and December 2020 were collected for this study, and 1,169 were screened for study inclusion. Of these patients, 650 were assigned to the incomplete ERAS group, and 519 were assigned to the non-ERAS group. After PSM (propensity score matching), 464 pairs of well-matched patients were generated for analysis.

Data from 1,258 patients were collected, and 1,169 were screened for study inclusion. Of these patients, 650 were assigned to the incomplete ERAS group, and 519 were assigned to the non-ERAS group (Figure 1), which had unbalanced clinical characteristics, such as age (*p* = 0.009), BMI (*p* = 0.009), ASA score (*p* < 0.001), tumor location (*p* = 0.020), neoadjuvant radiotherapy (*p* < 0.001), neoadjuvant chemotherapy (*p* = 0.003), and lymphovascular invasion (*p* < 0.001), as shown in Table 2. After propensity score matching, 464 pairs of well-matched patients were generated for comparative study.

### Postoperative complications

It can also be seen that after matching, patients in the incomplete ERAS group still had the advantage of fewer postoperative complications compared with those in the non-ERAS group (*p* = 0.002), as shown in Table 3. It can be seen from Table 4 that the incomplete ERAS protocol reduced the occurrence of postoperative complications (*p* = 0.008), among which the incidence of mild complications (Clavien–Dindo classification 1/2) was 6.7% vs. 10.8% (incomplete ERAS vs. non-ERAS), while the incidence of serious complications (Clavien–Dindo classification 3/4/5) was 3.2% vs. 6.0% (incomplete ERAS vs. non-ERAS). Then we used an alternative classification for complications: One is directly related to surgery, such as anastomotic leakage, surgical area infection, surgical area bleeding, celiac chylous fistula and urinary fistula. The other is the indirect complications of surgery, including respiratory system infection, delayed recovery of bowel function, arrhythmias, urinary tract infections, lower extremity venous thrombosis and anemia. Statistically,incomplete ERAS reduced indirect surgical complications (27,5.8% vs. 59, 12.7) but not direct complications (19,4.1% vs. 19, 4.1%).

**Table 3:** Characteristics of the patients after matching according to treatment group.

	Incomplete ERAS (*n* = 464)		Non-ERAS (*n* = 464)		*p*
	*N*	%	*N*	%	
Compliance with ERAS (midian + IQR^&^)	50% (45%–55%)				
Age	59.9 ± 11.8		59.7 ± 12.7		0.795
BMI	22.8 ± 3.0		22.9 ± 3.3		0.699
Tumor size	4.3 ± 2.3		4.3 ± 1.8		0.953
Sex					0.533
Male	311	67.0	302	65.1	
Female	153	33.0	162	34.9	
Fundamental disease					0.793
Yes	225	48.5	229	49.4	
No	239	51.5	235	50.6	
ASA score					0.700
1/2	433	93.3	430	92.7	
3/4	31	6.7	34	7.3	
Tumor location					0.121
1/2	98	21.1	107	23.1	
3/4	142	30.6	114	24.6	
5	224	48.3	243	52.4	
Neoadjuvant radiotherapy					1.000
Yes	25	5.4	25	5.4	
No	439	94.6	439	94.6	
Neoadjuvant chemotherapy					0.918
Yes	54	11.6	53	11.4	
No	410	88.4	411	88.6	
Differentiation					0.445
Low	49	10.6	59	12.7	
Moderate	399	86.0	385	83.0	
High	16	3.4	20	4.3	
Perineural invasion					0.398
Yes	91	19.6	81	17.5	
No	373	80.4	383	82.5	
Lymphovascular invasion					0.478
Yes	149	32.1	139	30.0	
No	315	67.9	325	70.0	
TNM stage					0.603
0	9	1.9	7	1.5	
1	73	15.7	74	15.9	
2	157	33.8	152	32.8	
3	171	36.9	189	40.7	
4	54	11.6	42	9.1	
Postoperative complications					0.002
Yes	46	9.9	78	16.8	
No	418	90.1	386	83.2	

IQR, interquartile range.

**Table 4:** The effects of incomplete ERAS on postoperative complications.

Clavien–Dindo Classification^a^	Incomplete ERAS (*n* = 464)		Non-ERAS (*n* = 464)		*p*
	*N*	%	*N*	%	
0	418	90.1	386	83.2	0.008
1/2	31	6.7	50	10.8	
3/4/5	15	3.2	28	6.0	
0	418	90.1	386	83.2	0.001
Local complications^b^	19	4.1	19	4.1	
Other Complications^c^	27	5.8	59	12.7	

^a^ Here, for statistical analysis purposes, complications are classified as mild (Clavien–Dindo 1 and 2) and severe (Clavien–Dindo 3, 4 and 5).

^b^ Complications directly related to the operation included anastomotic leakage, surgical area infection, surgical area bleeding, celiac chylous fistula and urinary fistula.

^c^ Other complications include respiratory system infection, delayed recovery of bowel function, arrhythmias, urinary tract infections, lower extremity venous thrombosis and anemia.

### Subgroup analysis of postoperative complications

To identify who might benefit from the incomplete ERAS protocol, different clinical characteristics of the two groups were subjected to subgroup analyses, as shown in Table 5. The subgroup analysis of postoperative complications revealed that all patients benefited regardless of sex (male, *p* = 0.037,11.9% vs. 17.9%; female, *p* = 0.010, 5.9% vs. 14.8%) and whether neoadjuvant chemotherapy was treated (neoadjuvant chemotherapy, *p* = 0.015, 7.4% vs. 24.5%; no neoadjuvant chemotherapy, *p* = 0.018,10.2% vs. 15.8%). However, subgroup analysis of other factors showed different results. Younger patients (<60 year, *p* = 0.002, 7.6% vs. 17.5%) had an advantage over older patients (≥60 year, *p* = 0.187, 12.0% vs. 16.2%). Patients with a low BMI (<22.84, *p* < 0.001, 9.4% vs. 21.1%) received a significant benefit, while those with a high BMI (≥22.84, *p* = 0.463, 10.5% vs. 12.7%) did not. Patients with a smaller tumor size (<4.0 cm, *p* = 0.004, 8.1% vs. 18.1%) were better than those with a larger tumor size (≥4.0 cm, *p* = 0.103, 11.3% vs. 16.0%). There was a significant difference in the incidence of complications between the two groups among those without fundamental diseases (*p* = 0.007, 8.8% vs. 17.0%), but the same result was not found among patients with fundamental diseases (*p* = 0.091, 11.1% vs. 16.6%). Patients with a low ASA score (1/2, *p* = 0.004, 9.7% vs. 16.3%) were more likely to benefit from the incomplete ERAS protocol, while no significant differences were found for patients with high scores (3/4, *p* = 0.270, 12.9% vs. 23.5%). A statistically significant difference was observed for patients with proximal colon tumors (ascending/transverse colon, *p* = 0.027, 12.2% vs. 24.3%), but not for patients with distal colon tumors (descending/sigmoid colon, *p* = 0.104, 7.7% vs. 14.0%) and rectal tumors (*p* = 0.140, 10.3% vs. 14.8%). Subgroup analysis of differentiation status showed that patients with poor (*p* = 0.012, 6.1% vs. 23.7%) and moderate (*p* = 0.034, 10.3% vs. 15.3%) differentiation in the incomplete ERAS group had fewer postoperative complications than those in the non-ERAS group. However, for the patients with high differentiation (*p* = 0.426, 12.5% vs. 25.0%), no statistically significant difference was found between the two groups. Patients without neoadjuvant radiotherapy significantly benefited from an incomplete ERAS protocol and had fewer postoperative complications (*p* = 0.004, 10.3% vs. 16.9%), whereas patients who received neoadjuvant radiotherapy did not benefit (*p* = 0.349, 4.0% vs. 16.0%).

**Table 5:** Subgroup analysis of postoperative complications.

	Incomplete ERAS (*n* = 464)		Non-ERAS (*n* = 464)		*p*	OR	95% CI
	*N* ^b^	%	*N* ^b^	%			
Sex							
Male (*n* = 613)	37	11.9	54	17.9	0.037	0.620	0.395–0.975
Female (*n* = 315)	9	5.9	24	14.8	0.010	0.359	0.161–0.801
Age (60 year)							
<median (*n* = 440)	17	7.6	38	17.5	0.002	0.389	0.212–0.713
≥median (*n* = 488)	29	12.0	40	16.2	0.187	0.708	0.423–1.185
BMI (22.84)							
<median (*n* = 462)	22	9.4	48	21.1	<0.001	0.385	0.224–0.663
≥median (*n* = 466)	24	10.5	30	12.7	0.463	0.808	0.457–1.429
Tumor size (4.0 cm)							
<median (*n* = 369)	16	8.1	31	18.1	0.004	0.397	0.209–0.755
≥median (*n* = 559)	30	11.3	47	16.0	0.103	0.665	0.407–1.088
Fundamental disease							
Yes (*n* = 454)	25	11.1	38	16.6	0.091	0.628	0.365–1.081
No (*n* = 474)	21	8.8	40	17.0	0.007	0.470	0.268–0.824
ASA score							
1/2 (*n* = 863)	42	9.7	70	16.3	0.004	0.552	0.367-0.831
3/4 (*n* = 65)	4	12.9	8	23.5	0.270	0.481	0.129–1.794
Tumor location							
1/2 (*n* = 205)	12	12.2	26	24.3	0.027	0.435	0.206–0.919
3/4 (*n* = 256)	11	7.7	16	14.0	0.104	0.514	0.229–1.157
5 (*n* = 467)	23	10.3	36	14.8	0.140	0.658	0.377–1.150
Differentiation							
Low (*n* = 108)	3	6.1	14	23.7	0.012	0.210	0.056–0.779
Moderate (*n* = 784)	41	10.3	59	15.3	0.034	0.633	0.413–0.969
High (*n* = 36)	2	12.5	5	25.0	0.426^a^	0.429	0.071–2.578
Neoadjuvant radiotherapy							
Yes (*n* = 50)	1	4.0	4	16.0	0.349^a^	0.219	0.023–2.114
No (*n* = 878)	45	10.3	74	16.9	0.004	0.563	0.379–0.838
Neoadjuvant chemotherapy							
Yes (*n* = 107)	4	7.4	13	24.5	0.015	0.246	0.074–0.813
No (*n* = 821)	42	10.2	65	15.8	0.018	0.608	0.401–0.920

^a^ Fisher's exact test.

^b^ *N*, number of patients with complications.

## Discussion

There have been many studies on the effects of ERAS on colorectal cancer surgery, and the vast majority of those studies have focused on the perioperative effects of highly compliant ERAS and the differences between ERAS and traditional care (21–24). However, in clinical practice, overall compliance with the ERAS protocol varies from hospital to hospital and depends on the hospital's teamwork, the medical staff's acceptance of the protocol (25), and patient cooperation. The perioperative benefits of complete ERAS do not need to be confirmed by more studies, according to Dr. Kehlet H., the proposer of the ERAS protocol, and the core elements of ERAS should be clarified for further improvement (26). Looking for such key factors in patients with lower ERAS compliance seems to be one possible route. To this end, we focused our research on exploring the perioperative complications of colorectal cancer patients who had low compliance with the ERAS protocol. To the best of our knowledge, to date, there are no large-scale comparisons of perioperative complications between incomplete ERAS and non-ERAS patients.

PSM is a statistical analysis method used to reduce the impact of data bias and confounding variables. In this study, we used PSM to balance the two sets of data. After PSM, the following imbalanced factors were well balanced: age (*p* = 0.795), BMI (*p* = 0.699), ASA score (*p* = 0.700), tumor location (*p* = 0.121), neoadjuvant radiotherapy (*p* = 1.000), neoadjuvant chemotherapy (*p* = 0.795), and lymphovascular invasion (*p* = 0.398). To better match the scoring and guide clinical practice, we regrouped patients according to the ASA score and tumor location. For ASA score, patients were classified according to a score of 1/2 and 3/4. For tumor location, patients were classified into the proximal colon group (including ascending colon/transverse colon) and distal colon (including descending colon and sigmoid colon) and rectum group according to the actual surgical judgment. Interestingly, with or without PSM, the NR group showed fewer postoperative complications than the conventional care group, but after PSM, the two groups were more comparable, as shown in Tables 2, 3.

Here, for statistical analysis purposes, complications are classified as mild (Clavien–Dindo 1 and 2) and severe (Clavien–Dindo 3, 4 and 5). Further analysis of the postoperative complications showed that even without high compliance to the ERAS protocol, the program still reduced the incidence of postoperative complications compared to traditional care, with fewer mild complications (6.7% vs. 10.8%) and severe complications (3.2% vs. 6.0%) observed. Fewer complications indirectly related to surgery were observed in the incomplete ERAS group, and as most surgeons know, Local surgical complications were related to presence of drain, the depth of the tumor, including T4b, operation time, and blood loss. This result suggests that ERAS affects other aspects of recovery, rather than the surgery itself. Based on this result, it is reasonable to further ask whether some elements in ERAS protocols are more clinically significant protective factors than others. Our study seems to serve as a support for such investigations, and our next step is to identify the key elements of ERAS and make the ERAS protocol easier to implement to help provide patients with better perioperative recovery.

In the sex subgroup analysis, the incomplete ERAS group had fewer complications than the non-ERAS group, regardless of sex (male, OR 0.620, 95% CI, 0.395–0.975; female, OR 0.359, 95% CI, 0.161–0.801). Similar results were observed in the neoadjuvant chemotherapy subgroup analysis. Thus, it was not deemed a factor that affected the reduction in perioperative complications of ERAS (neoadjuvant chemotherapy, OR 0.246, 95% CI, 0.074–0.813; no neoadjuvant chemotherapy, OR 0.608, 95% CI, 0.401–0.920). Although the OR values here indicate that neoadjuvant therapy is advantageous, this advantage should be considered with caution due to the small number of cases in this group. However, the subgroup analysis of several other factors presented biased options. The incomplete ERAS protocol was found to have a significant advantage for younger patients (<60 years, OR 0.389, 95% CI, 0.212–0.713) with a low BMI (<22.48,OR 0.385,95% CI, 0.224–0.663), small tumor size (<4.0 cm, OR 0.397,95% CI, 0.209–0.755), no fundamental diseases (OR 0.470, 95% CI, 0.268–0.824), low ASA score (OR 0.552, 95% CI, 0.367–0.831), ascending/transverse colon tumor (OR 0.435, 95% CI, 0.206–0.919), poor differentiation (OR 0.210, 95% CI, 0.056–0.779) or moderate differentiation (OR 0.633,95% CI, 0.413–0.969), and no neoadjuvant radiotherapy (OR 0.563, 95% CI, 0.379–0.838). In the statistical analysis, due to the small number of patients in the highly differentiated and neoadjuvant radiotherapy subgroups, Fisher's exact test was adopted to obtain more accurate results.

Based on these results, we believe it would be beneficial to attempt the ERAS protocol in as many patients as possible, especially young patients with a low BMI, small tumor size, low ASA score, proximal colon, poor/moderately differentiated tumor, and without fundamental diseases or neoadjuvant radiotherapy, because a reduction in postoperative complications, both mild and severe, was noted in this population. It is well known that patients with younger age, lower BMI, lower ASA, and no fundamental disease have a lower risk of complications. As to why such patients are more likely to benefit from ERAS programs, based on our experience, we believe that such patients are more likely to receive ERAS procedures and have better cooperation than older and frail patients who have more trouble getting postoperative ambulation early. The incidence of postoperative complications, surgical readmission or death within 30 days of surgery is extremely important for both health care workers and patients because more complications mean a longer total hospital stay, higher medicare payments (27), and more human resources.

Considering the impact of the introduction time of laparoscopic colorectal surgery on surgical complications, we selected those cases that went beyond the learning curve as the study subjects. Since the introduction of laparoscopic sigmoid resection in 1993, Chinese doctors have gradually accepted and mastered the technique. After 2000, laparoscopic colorectal resection has been performed in many medical centers in China. The four centers involved in the study were all university hospitals, and surgeons were well beyond the learning curve and proficient in laparoscopic surgery before the earliest cases were included. The incidence of postoperative complications was similar among the participating institutions.

There are some limitations to the study. First, although this is a multicenter study, the biases associated with retrospective studies are inevitable, so the results may be statistically biased. Second, most of the non-ERAS data we collected were from before the publication of ERAS for colorectal cancer surgery in 2012, while almost all the data in the incomplete ERAS group were from after 2012. This may have led to bias in the statistical analysis, thus affecting the reliability of the results. Therefore, an RCT cohort study must be completed in the future before the results of this study can be extended to a larger, more complex population.

## Conclusions

An incomplete ERAS protocol reduces the incidence of postoperative complications compared to no ERAS protocol, especially for younger patients who have a low BMI, smaller tumor size, no fundamental diseases, low ASA score, proximal colon tumors, poor/moderate differentiation and no preoperative neoadjuvant radiotherapy. ERAS should be recommended to as many patients as possible, although some patients are unable to adhere well to the protocol. In the future, more core elements of ERAS need to be identified.

## Data Availability

The raw data supporting the conclusions of this article will be made available by the authors, without undue reservation.
